# Characteristic of the excretory system in *Cassida palaestina* Reiche, 1858 (Coleoptera: Chrysomelidae: Cassidinae)

**DOI:** 10.1007/s00709-025-02072-y

**Published:** 2025-05-06

**Authors:** Mesut Sirri, Damla Amutkan Mutlu

**Affiliations:** 1 Department of Plant and Animal Production, Vocational School of Kurtalan, Siirt University, Siirt, Türkiye; 2 Faculty of Science, Department of Biology, Gazi University, Ankara, Türkiye

**Keywords:** Cryptonephric Malpighian tubules, Morphology, Histology, *Cassida*, Coleoptera

## Abstract

The Malpighian tubules are well-known and studied as the principal excretory organs in most insects. They play a key role in the production of primary urine and osmoregulation. It works with the rectum while regulating the water and salt balance in the body. The distal ends of the tubules are found in contact with the wall of the rectum in insects that feed on dry substances or live in a nearly dry environment and therefore, need to retain water: that is an arrangement known as a cryptonephric system. In this study, *Cassida palaestina* Reiche, 1858 is a beetle species belonging to the order Coleoptera was used as material, and the morphological features of the Malpighian tubules of this species were examined using light microscopy and scanning electron microscopy. The four cryptonephric Malpighian tubules of *C. palaestina* are found at the junction of the midgut and hindgut. The apical surface of tubule cells is surrounded by numerous microvilli. The cytoplasm of tubule cells fills with granules of many different sizes. Here, we reported our observations on the cryptonephridial complex in *C. palaestina*, and this study is almost the first study to examine the structure of the excretory system of the genus Cassida. Insights into the structure of the cryptonephridial complex of this species are compared with the well-studied cryptonephridial complexes of Cucujiformia. The findings were found to be quite similar to those of other species studied in the literature (with the structure of the Malpighian tubules of insects within the same order and from different orders). These data are the basis for future morphological studies. At the same time, the presence or absence of the cryptonephridial complex among species in the Cucujiformia infraorder, which *C. palaestina* is a part of, helps to understand the phylogenetic relationship.

## Introduction

The Malpighian tubule system is an excretory in insects. The system extends from the digestive canal and consists of branching tubules. The primary function is osmoregulatory. In addition to this function, tubules are responsible for the production of primary urine and the reabsorption of water, ions, and solutes (Farina et al. 2022; Chen et al. 2023). With the secretion of H +, K +, and Na + ions, water, electrolytes, and nitrogenous wastes pass from the hemolymph to the tubules. The tubule cells reabsorb water and beneficial substances, and the remaining waste materials are transferred to the lumen of the hindgut. More reabsorption is continued by the hindgut epithelial cells (Ruiz-Sanchez et al. 2007; Gautam and Tapadia 2014; Silva et al. 2019; Arab and Caetano 2002).

*Cassida palaestina* is a species from the family Chrysomelidae. Larvae and adults of this species live on the leaves. The host range of this species is quite wide. They are generally species such as *Carthamus tinctorius*, *Centaurea behen*, *Cynara scolymus*, and *Silybum marianum*, which belong to the Asteraceae family (Świętojańska et al. 2013). Nevertheless, it has been reported that it was collected from *Convolvulus arvensis*, *Amygdalus communis*, *Amaranthus ascendens*, *A. mangostanus*, *Atriplex nitans*, *A. patula*, *A. hastatum*, *Beta vulgaris*, *Chenopodium album*, *C. rubrum*, *C. glaucum*, *C. bonus-henricus*, *C. urbicum*, *C. polyspermum*, *C. vulgaria*, *Mentha* spp., and *Zea* spp. (Kısmalı and Sassi 1994; Borowiec and Świętojańska 1999; Çam and Atay 2004; Bolu 2016). The general distribution of this species is Afghanistan, Armenia, Cyprus, Greece, Iran, Iraq, Israel, Kazakhstan, Kyrgyzstan, Lebanon, Syria, Tadzhikistan, Turkey, and Turkmenistan (Borowiec and Ghahari 2021). Taxonomy and systematics of this species have been reported elsewhere (Ivie 1985; Borowski 2020; Güven et al. 2024). Güven et al. (2024) were stated that *C. palaestina* was detected for the first time in field studies conducted in different locations in the Eastern Anatolia Region. Borowski (2020) conducted a study on the distribution inventory of insects of the family Chrysomelidae (Coleoptera) in Eastern Europe and Northern Asia. Ivie (1985) studied the phylogeny of species in the order Coleoptera in his doctoral thesis and revealed the relationship of close species in systematics based on the presence and absence of cryptonephric Malpighian tubules. However, the structure of Malpighian tubules of adult *C. palaestina* was not observed. For this purpose, Malpighian tubules of adult *C. palaestina* were examined to contribute to the better comprehension of morphology and anatomy of the excretory system of this species.

## Material and methods

Twenty adult *C. palaestina* species were collected alive from three different regions Yüksekova district, Şemdinli district, and Büyük Çiftlik town of Hakkari province, Türkiye. Asst. Prof. Dr. Mesut SIRRI (collector) permanently stores the specimens’ vouchers at the collections in the Department of Plant and Animal Production. The species identification was made using the identification keys by Asst. Prof. Dr. Mesut SIRRI (Siirt University, Vocational School of Kurtalan, Department of Plant and Animal Production) and Asst. Prof. Dr. Neslihan BAL (Gazi University, Faculty of Science, Department of Biology). The excretory systems of the species brought to the laboratory environment were dissected under a stereomicroscope and stocked in different fixatives for light and electron microscope experiments. For light microscopy, samples were fixed in 10% Formaldehyde. After washing and dehydration, the samples were embedded in paraffin. Then, the sections were stained with Hematoxylin and eosin (HE) and photographed. For scanning electron microscopy, samples were fixed in 5% Glutaraldehyde. After the dehydration process, the samples were dried in air. The gold-coated samples were examined and photographed under a scanning electron microscope (SEM).

## Results

**Fig. 1:**
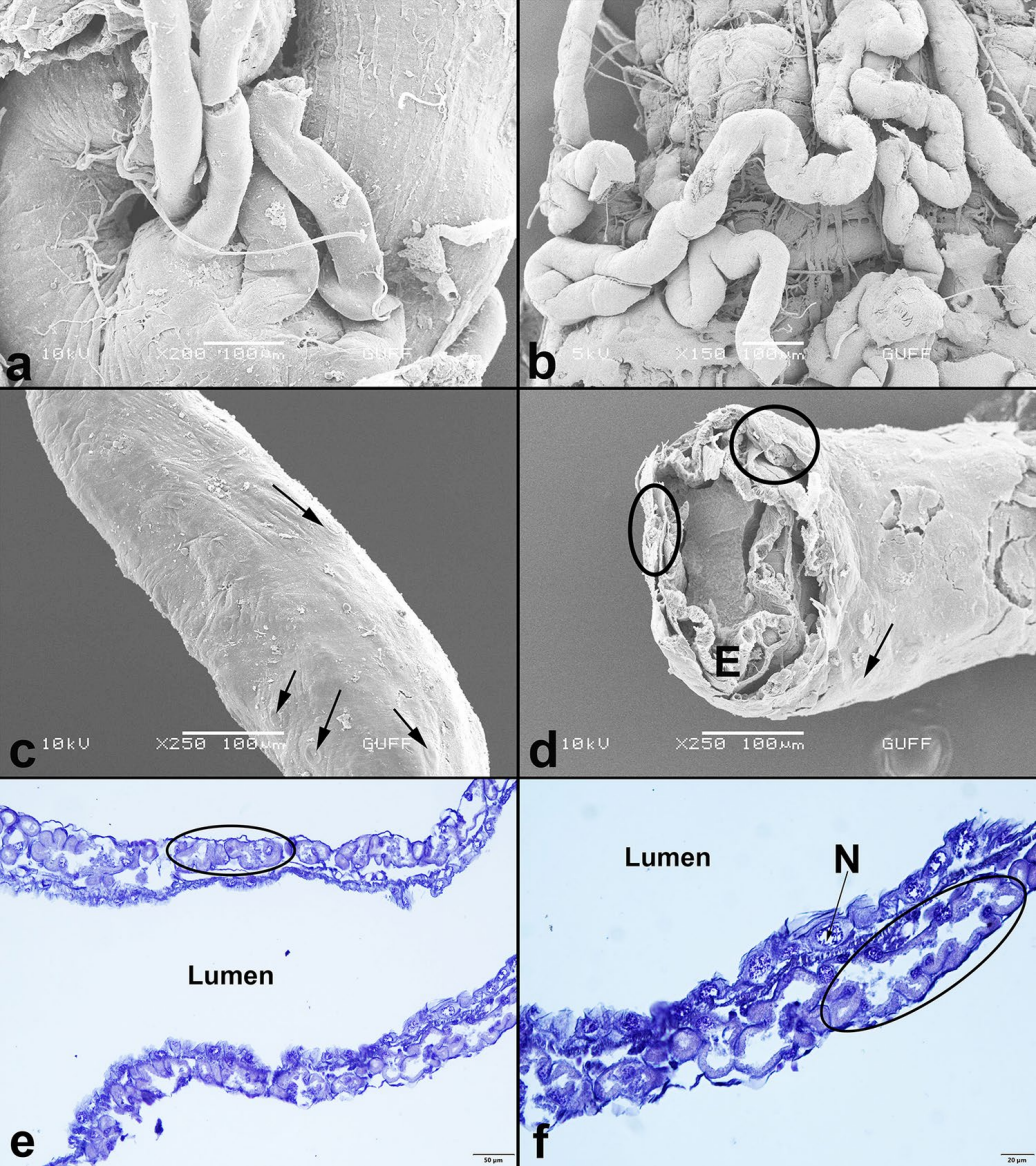
**a**-**b**. The general view of the Malpighian tubules in *Cassida palaestina*. The proximal tubules connect to the junction of the midgut and hindgut (SEM). **c.** The distal Malpighian tubules (→) attached to the colon (SEM). **d.** Circles shown that the distal Malpighian tubules under the sheath surrounding the colon (SEM). Colon epithelium cell (E). **e–f.** The longitudinal section of the distal Malpighian tubules (Circles) under the sheath surrounding the colon. Nucleus (N), (Light Microscope, HE)

**Fig. 2:**
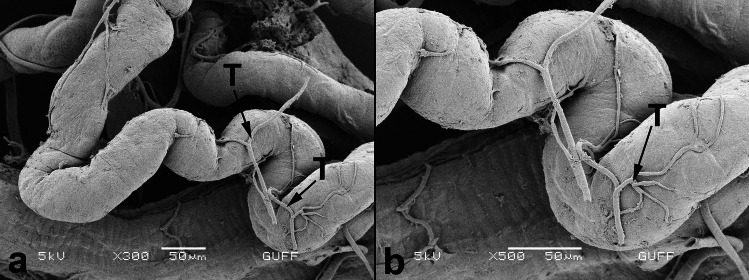
**a**-**b**. The outer surface of the Malpighian tubules in *Cassida palaestina* (SEM). Trachea (T)

**Fig. 3:**
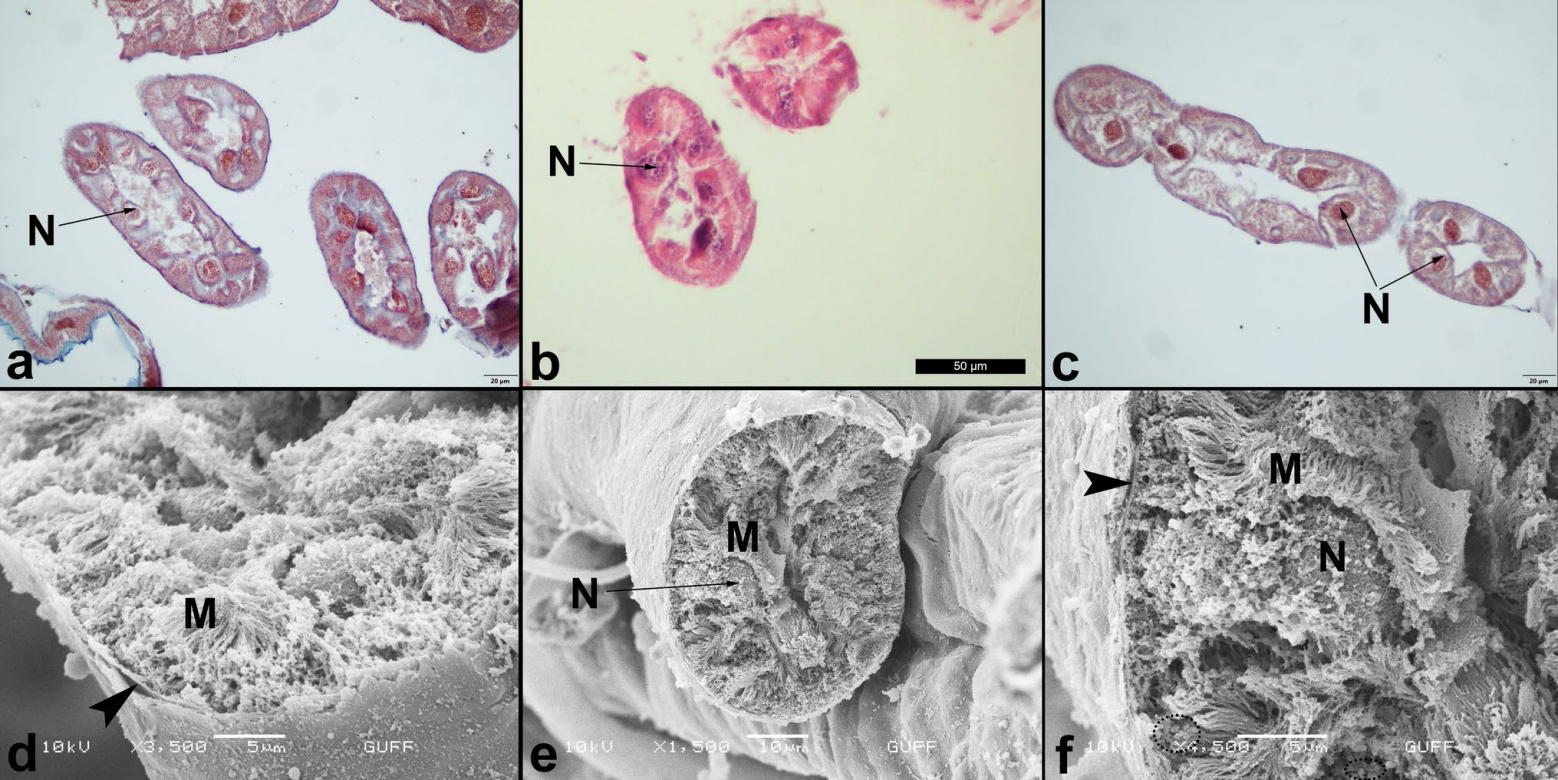
**a-c**. The cross section of the Malpighian tubules. Nucleus (N), (Light Microscope, HE). **d-f.** The cross sections of the Malpighian tubules in different magnifications in SEM. Microvilli (M), Arrowhead indicates thin basal lamina. Granules (◌)

Adults of *C. palaestina* have four slender Malpighian tubules, which are scattered all over the body cavity disorganized (Fig. 1A). The Malpighian tubules are located in the junction of the midgut and hindgut. The proximal ends of the tubules connect to the digestive system (Fig. 1A). It surrounds the digestive tract in places (Fig. 1B). Nonetheless, the distal ends of the tubules are in contact with the colon’s wall. The distal ends also form a layer under the sheath surrounding the colon (Fig. 1C). They are also distinguished from outside the sheath (Fig. 1D). This arrangement is known as cryptonephric system (Crowson 1981; Pacheco et al. 2014). The trachea and a thin muscle layer surround the surface of all tubules (Fig. 2A, B). Each Malpighian tubule comprises a single layer of cuboidal epithelium (Fig. 3A). In the images obtained, Malpighian tubules usually have between three and five epithelial cells (Fig. 3A). The thickness of tubule epithelial cells is on average 10 µm thick. A round nucleus is located in the middle of the cells (Fig. 3A-D). The cells rest on a thin basal lamina (Fig. 3B, D). There are small granules in the cytoplasm of the tubule cells (Fig. 3D). Cells have microvilli on their apical surfaces (Fig. 3B-D).

According to the data we obtained, no differences were observed in the morphological and histological features of Malpighian tubules between male and female individuals.

## Discussion

Malpighian tubules are the main excretory organs in insects. It has a connection to the digestive tract, which is the junction of the midgut, and the hindgut (Amutkan et al. 2015; Zhong et al. 2015; Amutkan Mutlu and Suludere 2020). Thus, waste materials are transferred to the digestive tract through the Malpighian tubules and thrown out of the hindgut (Gautam and Tapadia 2014). In some insects, the distal end of the Malpighian tubules is free in the hemolymph, while in some insects, it is in contact with the surface of the hindgut. The fact that the distal end of the Malpighian tubules is in contact with the surface of the hindgut is called the cryptonephridial system (Green 1980; Pacheco et al. 2014; Amutkan Mutlu and Suludere 2020). This system is found in the orders Coleoptera and Diptera (Green 1980; Amutkan Mutlu and Suludere 2020). Borges et al. (2015) stated that the rectum and the Malpighian tubules in *Adalia bipunctata* (Coleoptera, Coccinellidae) form a cryptonephric system. In another study, Aldigail et al. (2013) showed that there is a cryptonephric system at the junction of the colon and rectum, which corresponds to the beginning of distal Malpighian tubules in *Epilachna chrysomelina* (Coleoptera, Coccinellidae). Distal tubules in *Meligethes* (*Odonthogethes*) *chinensis* (Coleoptera: Nitidulidae) are attached to the colon, forming a cryptonephridial system. There is also a cryptonephridial system in the tubules of *C. palaestina*.

The tubules vary in number among different orders of insects. Most Coleoptera have four or six Malpighian tubules (Crowson 1981; Chen et al. 2023). A study showed that *Tribolium castaneum* (Coleoptera, Tenebrionidae) has six Malpighian tubules (King and Denholm 2014). Similarly, *M.* (*O.*) *chinensis* (Coleoptera: Nitidulidae) has six Malpighian tubules (Chen et al. 2023). While most Cleridae have six Malpighian tubules (Ekis and Gupta 1971), some species have four Malpighian tubules noted it. Another study stated that *Holitrica oblita* (Coleoptera, Melolonthidae) has four Malpighian tubules (Song et al. 2012). Hanrahan and Nicolson (1987) stated that there are six Malpighian tubules in *Onymacris plana plana* (Coleoptera, Tenebrionidae). Kasap (1975) indicated that there are six Malpighian tubules in *Cassida rubiginosa* (Coleoptera, Chrysomelidae), *Cassida flaveola* (Coleoptera, Chrysomelidae), *Cassida nebulosa* (Coleoptera, Chrysomelidae), and *Cassida viridis* (Coleoptera, Chrysomelidae). Similar features were noted when examining monophagous species from the Coleoptera order. Bu and Chen (2009) observed that six Malpighian tubules connect to the digestive tract in *Dendroctonus armandi* (Coleoptera, Scolytinae). It has been reported that there is a total of six cryptonephric Malpighian tubules in *D. micans* (Coleoptera, Scolytidae), *D. ponderosae* (Coleoptera, Scolytidae), *D. pseudotsugae* (Coleoptera, Scolytidae), *D. rufipennis* (Coleoptera, Scolytidae), and *D. terebrans* (Coleoptera, Scolytidae), with four tubules positioned anteriorly and two tubules positioned posteriorly (Díaz et al. 2003). There are three cryptonephric Malpighian tubes in *Hypocryphalus mangiferae* (Coleoptera, Scolytinae) (Serrão et al. 2008). When looking at other insect orders, the number of Malpighian tubules varies considerably. Tubule number is relatively high in species from the order Orthoptera. On the other hand, in species from the order Hemiptera, the number of tubules is at most four (Pacheco et al. 2014; Amutkan Mutlu and Suludere 2020). *C. palaestina* examined in this paper has four Malpighian tubules like the order in which it is found.

Active cells in the Malpighian tubules of insects are characterized by long and numerous microvilli and the presence of spherical granules in the cell cytoplasm (Gonçalves et al. 2018). These granules are also called spherite or spherocrystal. These granules are known as mineral storage structures. These stored minerals may vary during the developmental process in insects (Ballan‐Dufrançais 2002; Gonçalves et al. 2018; Amutkan Mutlu and Suludere 2020). In *C. palaestina*, granules of small sizes were found in the tubule cell cytoplasm, similar to those in the Malpighian tubule cell cytoplasm of other insects.

Cucujiformia is the largest infraorder of Coleoptera. This infraorder is also characterized by including the vast majority of phytophagous beetles and have cryptonephric Malpighian tubules. The insects in this group are adapted to live in drier habitats or to spend more time on exposed parts of plants. This adaptation is due to the presence of cryptonephric Malpighian tubules, which results in an improved water reabsorption mechanism (Ivie 1985). Beaven and Denholm (2025) stated that the cryptonephric system in desert tenebrionid beetles is a necessary water conservation adaptation and therefore an ecological adaptation indicator. The loss of the cryptonephridial system in many aquatic species is viewed as an environmental adaptation that may have a primary function in water conservation in terrestrial species (Beaven and Denholm 2025). Cryptonephridism is known in all insects belonging to Cucujiformia, Bostrichiformia, Scarabaeoidea, and Buprestoidea (Crowson 1961; Lawrence and Hlavac 1979). A closer relationship between Bostrichiformia and Cucujiformia is closely related to the fact that they are higher phytophagous species and therefore have cryptonephric Malpighian tubules (Poll 1932; Stammer 1934; Saini 1964; Ivie 1985). Saini (1964) and Poll (1932) pointed out that the amount of water in the food content of insects is related to their possession of the cryptonephric Malpighian tubule. They therefore suggested the lack of cryptonephridism in aquatic cucujiforms. This supports the systematic closeness of species in the infraorder Bostrichiformia and Cucujiformia (Ivie 1985). Lyal and Favreau (2015) stated that the Cryptonephridial Malpighian tubule structure in various Coleoptera orders suggests it may aid in giving phylogenetic information to identify monophyletic groups.

This study presents the morphological features of Malpighian tubules of *C. palaestina*, becoming a resource for future studies on insects’ excretory system. More observations are needed to have more information about cryptonephridial Malpighian tubule morphology and to better understand the phylogenetic relationships between species.

## Declarations

The author is an Editorial Board Member/Editor-in-Chief/Associate Editor/Guest Editor for *[Journal name]* and was not involved in the editorial review or the decision to publish this article.
